# Quinoa for Marginal Environments: Toward Future Food and Nutritional Security in MENA and Central Asia Regions

**DOI:** 10.3389/fpls.2016.00346

**Published:** 2016-03-29

**Authors:** Redouane Choukr-Allah, Nanduri K. Rao, Abdelaziz Hirich, Mohammad Shahid, Abdullah Alshankiti, Kristina Toderich, Shagufta Gill, Khalil Ur Rahman Butt

**Affiliations:** Research and Innovation Division, International Center for Biosaline AgricultureDubai, UAE

**Keywords:** irrigation, salinity, yield, sowing dates, food security

## Abstract

Quinoa is recognized as a crop of great value in terms of tolerance to abiotic stresses and there is growing interest to introduce it in marginal agricultural production systems worldwide. Also, quinoa is one of the most nutritious food crops currently known and the nutritive properties of the crop are seen as a mean to fight malnutrition globally. Various quinoa cultivars have been screened for tolerance to salinity, water-use efficiency and nutritional quality and the positive attributes found in them have created even wider global interest in its cultivation. This paper summarizes 15 years of studies on assessing the potential for introducing the crop in a few countries of the Middle East and North Africa (MENA) and Central Asia regions and describes the key constraints for scaling-up the production under marginal growing conditions in the newly introduced countries.

## Introduction

Quinoa has been recognized as a climate resilient crop of great value and there is an increasing effort to introduce it in different marginal agriculture production systems of the world. Various quinoa cultivars have been screened for tolerance to abiotic stresses, especially salinity, drought, and frost and the positive attributes of the crop have created wider global interest in its cultivation (Jacobsen, 2003; Jacobsen et al., 2003). Also, quinoa is one of the most nutritious food crops currently known. The seeds contains high quality protein, which has all of the essential amino acids including lysine, methionine and threonine that are scarce in cereals and legumes (Repo-Carrasco et al., 2003). In view of its exceptional nutritional quality and ability to grow under marginal environments, the Food and Agriculture Organization of the United Nations (FAO) has identified quinoa as one of the crops that will play an important role in ensuring future food security and designated the year 2013 as the “Year of Quinoa” (Bazile et al., 2015). Worldwide, the demand for quinoa is growing, especially in the health food segment, but current supplies are unable to match it. Besides the use for human consumption, quinoa seed has other uses as livestock and poultry feed. The whole plant can be used as green fodder and harvest residues can be fed to the animals. In the context of the Middle East and North Africa (MENA) and Central Asia regions, where soil and water salinity is increasingly becoming a constraint to agricultural production, quinoa is seen as an alternative crop with significant potential to have a central role in sustaining farm productivity.

The International Center for Biosaline Agriculture (ICBA) in Dubai, United Arab Emirates (UAE) has been working since 2007 on quinoa in partnership with the Ministry of Environment and Water (MOEW) of the UAE, Abu Dhabi Farmer's Service Center (ADFSC) and its Peruvian partners [Instituto Nacional de Innovacion Agraria (INIA) and Universidad National Agraria La Molina (UNALM)] to evaluate the performance of several quinoa cultivars for their productivity when grown in marginal conditions. ICBA has identified and developed five high yielding salt- and heat-tolerant lines that are now ready to be tested in other agro-ecological zones. The FAO has also been in the forefront of quinoa research, keeping in view its unique nutritional characteristics and adaptability to a wide range of agro-ecological conditions, especially in the marginal areas and the organization has initiated a Regional Project TCP/RAB/3403 for the introduction, appropriation and institutionalization of its production in Algeria, Egypt, Iran, Iraq, Lebanon, Mauritania, Sudan, and Yemen. ICBA considers quinoa as an essential crop in its efforts to sustain agricultural productivity amidst growing threat of salinity and water scarcity, as well as to tackle hunger, malnutrition and poverty in the MENA and Central Asia regions. To fully exploit the potential of the crop for marginal environments, identification of new and high-yielding quinoa genotypes with good local adaptation and high nutritional quality are crucial, which requires intensified screening and adaption research.

This paper summarizes 15 years of studies in assessing the potential of quinoa for introducing it in some countries of the MENA and Central Asia Regions. In view of quinoa's potential as a stress-tolerant, climate resilient future-proof crop, ICBA is developing best practices for its production and management in marginal environments. The Center is also focusing on utilization and marketing of the produce and on fostering knowledge transfer, strengthening competencies and exchange of experiences among the various stakeholders within the MENA and Central Asia regions.

## Screening for local adaptation and yield potential

ICBA acquired 121 germplasm accessions from the United States Department of Agriculture (USDA) and evaluated them for growth performance and yield at its research station (25°05′49″ N and 55°23′25″E) during the cropping season November 2006–March 2007. The soil at the experimental site was fine sand and moderately alkaline (pH 8.2) with very low organic matter (< 0.5%). The seeds were sown in the first week of November 2006 and each accession was planted in three 3 m-rows with spacing of 50 cm between the rows and one meter between two accessions. The distance between plants within each row was maintained at 25 cm. Before sowing, the fertility of the soil was improved by incorporating organic fertilizer (compost) at the rate of 40 t ha^−1^ and during crop growth two split doses of NPK (20:20:20) at the rate of 50 kg ha^−1^, were applied by banding alongside the rows. The amount of N added to the soil by the combination of compost and NPK was estimated to be 570 kg ha^−1^. The plants were irrigated with low-salinity water with an electrical conductivity (EC_*w*_) of 2–3 dS m^−1^ using the drip system. Water was applied once every day for 20 min at a flow rate of 4 l h^−1^ per plant and in addition, about 60 mm of rainfall was received during the growing season. Of the 121 accessions planted, seeds were harvested from 73 accessions that survived through the growing season and based on the seed yield data, the top 20 performers were selected for subsequent evaluation, carried out during the cropping seasons 2007-08 and 2008-09 (Rao and Shahid, 2012). The agronomic practices such as irrigation and applications of fertilizers were the same as described previously. The data on grain yield of the 20 accessions from the two cropping seasons (2007-08 and 2008-09) are presented in Table 1. The seed yield among the accessions varied between 53.86 and 359.86 g m^−2^ in 2007-08, and between 3.32 and 258.42 g m^−2^ in 2008-09. Averaged over the two seasons, the yield ranged between 34.06 g m^−2^ and 238.99 g m^−2^ among the accessions. The studies showed that quinoa has good adaptation and can be successfully cultivated in the Arabian Peninsula. In a further study conducted at the ICBA Research Station in Dubai during 2009-10, the performance of five top-yielding accessions selected on the basis of average seed yield from previous studies were further evaluated using low-salinity irrigation water (EC_w_ 2.8 dS m^−1^) (Rao and Shahid, 2012). Analysis of the data revealed significant differences for plant height, number of primary branches and number of inflorescences per plant. However, differences among accessions for all other traits including inflorescence length, seed yield per plant, fresh and dry biomass were found to be marginal. The seed yield among the five accessions ranged between 374.4 g m^−2^ (Ames 13757) and 533.6 g m^−2^ (Ames 13761) with an average of 456.6 g m^−2^ over accessions. The dry matter yield, averaged over the accessions was 1464 g m^−2^, with accession Ames 13742 producing the maximum yield of 1624 g m^−2^ (Rao and Shahid, 2012). During course of the yield trials over the years, mass selection to eliminate inferior plant types and improve the yield potential of the selected accessions has resulted in five improved lines (Q1–Q5) which were used in further studies described below.

**Table 1:** **Grain yields in 20 selected quinoa accessions grown in winter 2007-08 and 2008-09 (source: Rao and Shahid, 2012)**.

**Accession no**.	**Source**	**Yield (g m^−2^)**		
		**2007-08**	**2008-09**	**Mean**
Ames 13220	Bolivia	84.57	100.95	92.76
Ames 13719	USA	74.61	87.05	80.83
Ames 13723	USA	80.09	134.42	107.25
Ames 13724	USA	132.32	62.42	97.37
Ames 13727	USA	111.39	133.02	122.2
Ames 13736	USA	57.50	109.07	83.29
Ames 13742	USA	359.86	118.12	238.99
Ames 13749	USA	211.06	79.25	145.16
Ames 13757	USA	187.26	120.32	153.79
Ames 13758	USA	64.81	3.32	34.06
Ames 13761	USA	60.50	258.42	159.46
Ames 21931	Bolivia	84.19	39.88	62.04
Ames 22154	Chile	50.85	45.13	47.99
Ames 22155	Chile	147.34	91.43	119.39
Ames 22157	Chile	118.58	133.33	125.95
NSL 106395	USA	105.77	119.25	112.51
NSL 106398	USA	54.00	193.92	123.96
NSL 106399	USA	167.10	148.65	157.87
NSL 86649	USA	53.86	143.1	98.48
PI 478410	Bolivia	58.06	13.27	35.67
Mean		113.19	106.72	109.95
SE		17.79	14.40	16.12

## Agronomic evaluation and performance of selected lines

The results from the preliminary trials undertaken at ICBA Research Station gave sufficient indication of the potential of quinoa as a salt-tolerant alternative crop. However, further investigations were needed to study the performance under a range of biophysical environments especially at various soil and water salinities to introduce the crop to the farmers. Hence field trials were conducted under a range of agro-ecological conditions—at Ghayathi in the Western Region of Abu Dhabi Emirate in 2012-13 with two of the five selected lines (Q3, Q5) and NSL 106399; and subsequently in five locations—three in the Northern Emirates (Dibba, Hamraniah, and Al Dhaid) and two in the Western Region of Abu Dhabi (Madinat Zayed and Ghayathi) in 2013-14, involving four of the five selected quinoa lines (Q1, Q2, Q3, and Q5) (Table 2). While the two locations planted in the Western Region were agricultural farms leased for the trials, those in the Northern Emirates were the federal government's regional research stations. Each set of lines was sown in a randomized complete block design (RCBD) with three replications. In all cases, the distance between rows was 50 cm and between plants within the row was 25 cm. Spacing between two adjacent plots was maintained at 1 m. The amount of water and fertilizers applied were the same for all the five locations and as described in the previous section.

**Table 2:** **Quinoa yield trails in the UAE: Soil and water characteristics at the locations and details of accessions evaluated (Rao, 2016)**.

**Location**	**Growing season**	**Soil texture**	**Water salinity (dS m^−1^)**	**Identity**
ICBA Reserach Station	2009-10	Sand	2.8	Ames 13742, Ames 13749, Ames 13757, Ames 13761, NSL 106399
Ghayathi	2012-13	Sand	15.1	ICBA-Q3, ICBA-Q5, NSL 106399
Ghayathi	2013-14	Sand	16.3	ICBA-Q1, Q3, Q4, Q5
Madinat Zayed	2013-14	Sandy loam	18.9	ICBA-Q1, Q3, Q4, Q5
Dibba	2013-14	Sand	6.1	ICBA-Q1, Q3, Q4, Q5
Hamraniah	2013-14	Sandy loam	4.5	ICBA-Q1, Q3, Q4, Q5
Al Dhaid	2013-14	Loamy sand	2.3	ICBA-Q1, Q3, Q4, Q5

**Figure 1:**
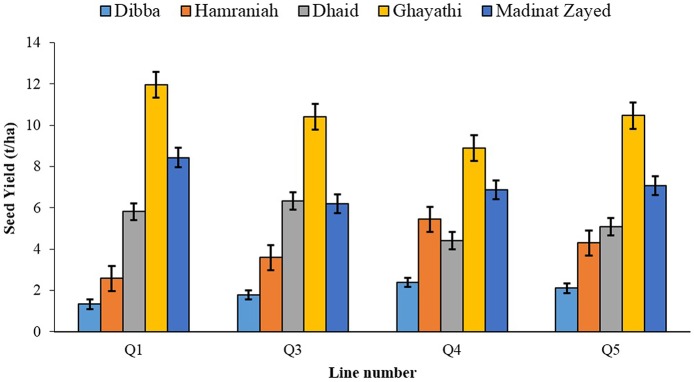
**The mean seed yields of four quinoa lines grown at five locations in the UAE during 2013-14**. The error bars represent LSD of the means (*p* < 0.05) (Rao, 2016).

In 2012-13, at Ghayathi, despite high salinity of irrigation water (EC_w_14-15 dS m^−1^), the mean seed yield obtained of the three lines was 750 g m^−2^ which was on par with the highest yields reported from the non-saline traditional quinoa growing areas. The green biomass yield was also high, the mean of the three cultivars being 4.3 kg m^−2^, indicating the potential of quinoa as an alternative forage crop for saline areas (Rao et al., 2013). In 2013-14 trials, the salinity of irrigation water in the five locations varied between 2.3 and 18.9 dS m^−1^ (Table 2). Seed yield from trials in the Northern Emirates correlated with the salinity of irrigation water (EC_*w*_), which was 6.1 dS m^−1^ in Dibba, 4.5 dS m^−1^ in Hamraniah and 2.3 dS m^−1^ in Al Dhaid. Thus, averaged over lines, the seed yield was highest in Al Dhaid (541 g m^2^), followed by Hamraniah (398 g m^−2^) and Dibba (190 g m^2^) (Rao, 2016; Figure 1). In the western region despite higher salinity of the irrigation water, significantly higher seed yields were recorded than those from the Northern Emirates. In Ghayathi, the mean seed yields of the four lines was 1050 g m^−2^, much higher than the yields recorded in the previous year (750 g m^−2^) from the same location. In Madinat Zayed the mean seed yield of the four lines was 700 g m^−2^, which was similar to the yield obtained from Ghayathi in the previous year (Rao et al., 2013; Rao, 2016; Figure 1). The results from yield trials showed high degree of variability in the performance of the different cultivars across locations (Supplementary Figure 1). While no major differences existed among the different locations in terms of radiation, mean maximum and minimum temperatures or precipitation during the growing period, the differences in yields could only be attributed to differences in physicochemical properties of the soils and water (other than the EC and pH which were measured), for which more detailed analyses are warranted. The two locations planted in the Western Region being agricultural soils, possibly had a more favorable microenvironment compared to the sandy soils with poorly developed profiles at the research stations in Northern Emirates. Nevertheless, the exceptionally higher yields obtained with highly saline irrigation water in the Western Region showed that quinoa, which is a facultative halophyte, not only endures salinity but also some of its cultivars prosper under saline conditions, therefore has huge potential as an alternative food and feed crop when growing traditional crops becomes uneconomical due to increased groundwater salinity. The results besides confirming quinoa's suitability to withstand high salinity in water and soils, also demonstrated that it has good adaptation to the hyper-arid desert environments and is an excellent candidate for crop diversification in the UAE as well as other countries with similar climatic conditions.

**Figure 2:**
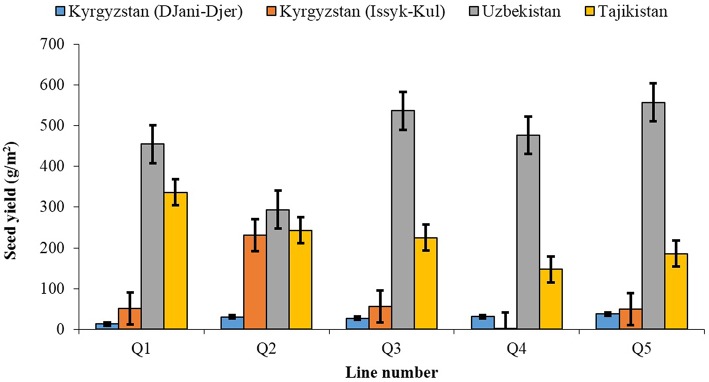
**The mean seed yields of five quinoa lines grown at four locations in Central Asia**. The error bars represent standard errors of the means (ICBA, 2015).

In addition to the UAE, ICBA has recently undertook pilot trials in six other MENA and Central Asian countries and the selected quinoa lines proved to be very productive even under very poor soils (sandy soils) and under conditions of deficit irrigation and high salinity. For instance, in 2015, five quinoa lines (Q1–Q5) were evaluated for seed and forage yields in three countries of Central Asia, namely: Uzbekistan, Tajikistan and Kyrgyzstan under different eco-agroclimatic zones that significantly differ in soil characteristics and agricultural practices (ICBA, 2015). Results from Kyrgyzstan were variable due to late planning but in Uzbekistan the seed yields among the lines ranged between 294 g m^−2^ (Q3) and 557 g m^−2^ (Q5) with a mean of 436 g m^−2^ and in Tajikistan they ranged from 147 g m^−2^ (Q2) to 336 g m^−2^ (Q1) with a mean of 227 g m^−2^ (Figure 2).

The high yields obtained under marginal agro-climatic conditions in different locations are indicative of the potential and further investigations are needed to study the performance of a much wider range of genetically diverse accessions at various soil and water salinities to fully exploit the available genetic diversity within the crop. Quinoa is a predominantly self-pollinating species and considerable variation exists between cultivars for many of the desired characters. Therefore, it should be possible to select better adapted genotypes with high yields and nutritional quality combined with salt-and drought-tolerance. Identification of desirable genotypes needs to be followed by work on optimization of cultural practices to maximize productivity under the local conditions.

**Figure 3:**
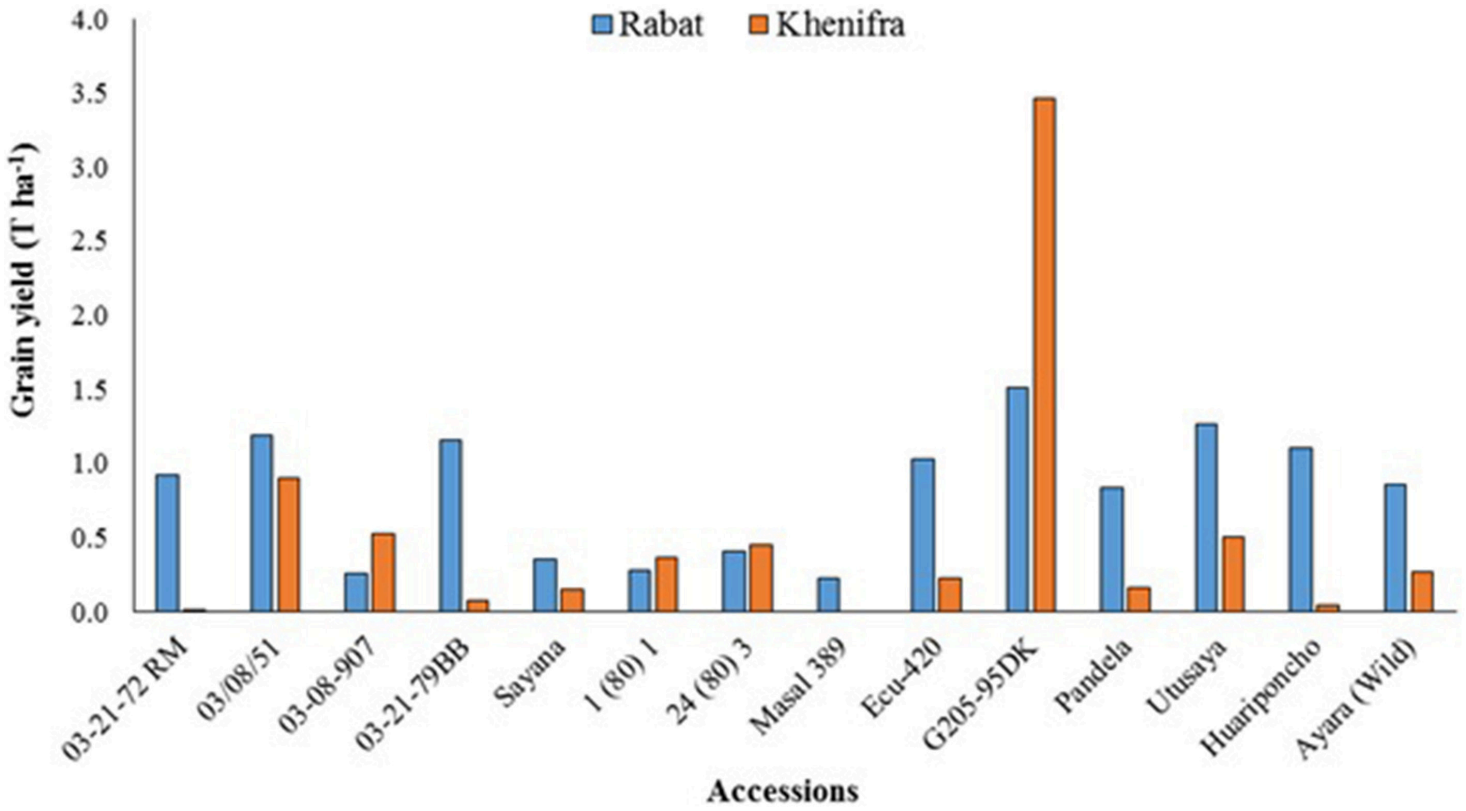
**Quinoa grain yields (T ha^−1^) under high altitude (Khenifra) and coastal (Rabat) conditions in 2000-01 season (Benlhabib et al., 2015)**.

Quinoa was evaluated and introduced in Morocco for the first time in 2000-01 growing season in two locations, Khenifra (mountain region) and Rabat (coastal region). Obtained results are presented in Figure 3. Among the tested lines, G205-95DK performed the best under high altitude conditions due to its origin in the high altitudes of Altiplano (Andean Plateau), while most of the other lines showed adaptation to the coastal region probably because of provenance effect (Benlhabib et al., 2015).

**Figure 4:**
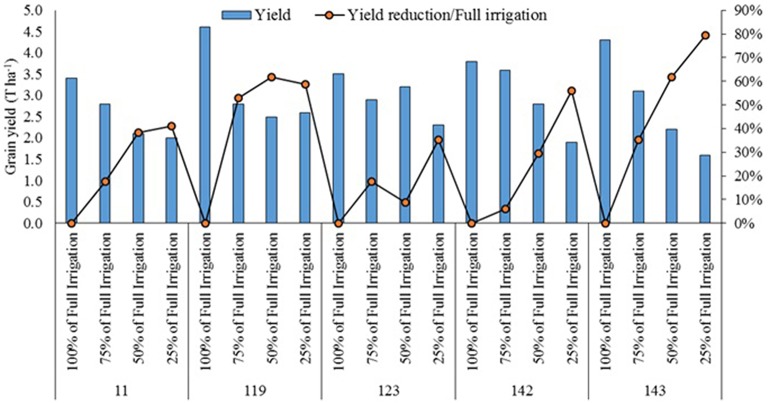
**Responses of 5 quinoa lines to different irrigation levels in terms of yield (Filali, 2011)**.

In Morocco, in a field experiment conducted in Rhamna region, four selected quinoa lines (L11, L119, L123, L142, L143) along with two commercial varieties Titicaca and Puno were evaluated for phenological and agro-morphological characteristics (Filali, 2011). Titicaca and L143 recorded the highest seed yield (1.5 t ha^−1^), while L11 showed the lowest seed yield (0.46 t ha^−1^). The same lines when cultivated in the experimental farm of the Agronomic and Veterinary Medicine Hassan II Institute, Horticultural complex, Agadir (IAV-CHA) and tested under four different irrigation levels (100, 75, 50, and 25% of full irrigation), responded differently to water stress (Figure 4). The line L143 was most affected by water stress, L123 and L11 were less affected as the reduction in yield compared to the fully irrigated treatment was less (Supplementary Figures 5, 6).

## Nutritional quality

Quinoa seeds are an exceptionally nutritious food source, owing to their high protein content with all essential amino acids, lack of gluten, and high content of several minerals such as Ca, Mg, Fe, and it is also rich in vitamins. Abiotic stress is known to induce considerable changes in the composition and quality of cereal grains such as wheat (Ashraf, 2014). Despite quinoa's growing popularity, the knowledge about the relationship between the growth conditions and the nutritional profile is still limited. Characterization of the nutritional and anti-nutritional properties of quinoa and evaluation of the effects of extreme growing conditions on the nutritional quality will provide strategic information for the introduction and promotion of quinoa in new environments besides aiding in the selection of nutritionally high and stable cultivars. Results from analysis of the chemical composition of the seeds harvested from three locations (Al Dahid, Ghayathi and Madinat Zayed) with varying levels of irrigation water salinity (2.3, 16.3, and 18.9 dS m^−1^, respectively) showed that salt stress has marginal effect on the proximate composition (Rao, 2016). However, significant differences were found in the mineral content of the seeds. Especially, seeds harvested from Ghayathi and Madina zayed had higher Na content, but lower levels of Ca and Fe compared to those harvested from Al Dhaid (Rao, 2016). These findings have significant implications, especially for introduction of quinoa in highly saline areas, therefore detailed investigations are recommended for confirmation.

## Agronomic trials and cultivation

In the FAO's Regional Project TCP/RAB/3403, 11 quinoa cultivars were evaluated for seed yield potential in seven of the eight participating countries (Egypt, Iraq, Iran, Lebanon, Mauritania, Sudan and Yemen) during January-June 2014. Large yield variations per hectare were observed across locations within and between countries, the mean seed yields ranging from 0.11–0.96 t ha^−1^ in Iraq, 0.24–1.90 t ha^−1^ in Yemen, 0.41–3.87 t ha^−1^ in Egypt, 1.50–7.50 t ha^−1^ in Lebanon, 0.16–1.56 t ha^−1^ in Iran, and 0.03–0.23 t ha^−1^ in Mauritania. The highest yield (7.50 t ha^−1^) was recorded in Lebanon, followed by Egypt (3.87 t ha^−1^), while the lowest yield was recorded in Mauritania (0.23 t ha^−1^) (Dost, 2015). A comparison of the cultivars across the countries/locations revealed very interesting and consistent patterns of performance that resulted in the identification of several potential/suitable varieties for the countries. The cultivars Q12 and Q21 exhibited outstanding performance in almost all the countries of evaluation, while Q29, Q18, Q19, Q22, Q26, and Q27 produced superior yields as compared to other cultivars at all locations/countries except Iran. Convinced by quinoa's performance, one private agriculture company in Egypt and a farmer in Lebanon planted quinoa on a large-scale (1–2 hectares) with a plan to further extend the production together with improvement in capacity of farmers and generating market demand. However, private sector involvement in quinoa production will mainly depend on the conducive policies of the governments, allocation of financial and human resources for research and development of new varieties along with the improvement in the production technologies, processing and utilization within the region.

## Cropping practices of quinoa production

### Deficit irrigation

In quinoa, deficit irrigation (DI) strategy has been widely investigated as a valuable and sustainable production strategy in regions where intra-seasonal dry spells are of considerable importance (Garcia et al., 2003; Geerts et al., 2008). By limiting water applications to drought sensitive growth stages, the practice aims to maximize water productivity and to stabilize, rather than maximize yields (Geerts and Raes, 2009). Several experiments were conducted at IAV-CHA during two growing seasons 2010 and 2011 in order to evaluate the effect of deficit irrigation with treated wastewater (Hirich et al., 2012a,b,c, 2013). During the first season (2010), the response of the line D0708 with six deficit irrigation treatments (during vegetative growth, flowering, grain filling, during both vegetative growth and flowering stage, alternating with 100% of full irrigation as non-stress condition, and 50% of full irrigation as water deficit condition) were studied. During the second season (2011), two lines (D0708 and QM1113) were studied with six other irrigation treatments (rainfed, 0, 25, 50, 75, and 100% of full irrigation) only during the vegetative growth stage, while in the remaining stages, full irrigation was provided except for the rainfed treatment. The results clearly showed that the highest yields were obtained in the fully irrigated treatment followed by the treatment stressed during vegetative growth stage. When subjected to deficit irrigation during flowering, grain filling, during both vegetative growth and flowering, and during all crop stages, a yield reduction of 32, 37, 46, and 50% was recorded respectively, in comparison with the control (full irrigation). The finding indicates that applying deficit irrigation during vegetative growth stage results in increased root to shoot ratio. Early water deficit induced root development and full irrigation applied during flowering and grain filling allowed the plants to produce more biomass due to the well-developed root system, possibly leading to higher levels of water and nutrient uptake. The increased yield observed in the treatment stressed during vegetative growth stage is probably due to maximized crop water productivity (CWP), which further allowed up to 19% water saving compared to full irrigation.

Statistical analysis carried out on grain yield of quinoa in response to several degrees of water stress applied during the vegetative growth stage revealed highly significant differences among the treatments (*p* < 0.001). The results clearly showed that line DO708 showed the highest grain yield compared to QM1113. While applying 50% of full irrigation during the vegetative growth stage resulted in 10 and 8% increase in seed yield in DO708 and QM1113 respectively compared to control (fully irrigated), applying 25% of full irrigation caused a yield reduction of 31 and 38% (in DO708 and QM1113 respectively, compared to the control). It is obvious that CWP is maximized under 50% deficit irrigation during the vegetative growth stage in both the lines.

Results from field trials conducted in sandy soil during two growing seasons (2011 and 2012) at the Experimental Station of Cadi Ayyad University located in 70 km south West Marrakech (Morocco) and also under rainfed condition in a farmer's field at Tnin Bouchan, about 250 km away from Agadir indicated that deficit irrigation (50 and 33% of full irrigation) affected quinoa performance and resulted in seed yield reduction of 15.8, 30.1% respectively in the first season (2011) and by 15.2, 41.5% respectively, in the second season (2012) in comparison with full irrigation. Under rainfed conditions, seed yields were reduced by 62.1 and 59.3% in the 2011 and 2012 growing seasons, respectively compared to full irrigation. CWP was maximized in the treatment receiving 50% of full irrigation (Fghire et al., 2013).

### Organic amendment

**Figure 5:**
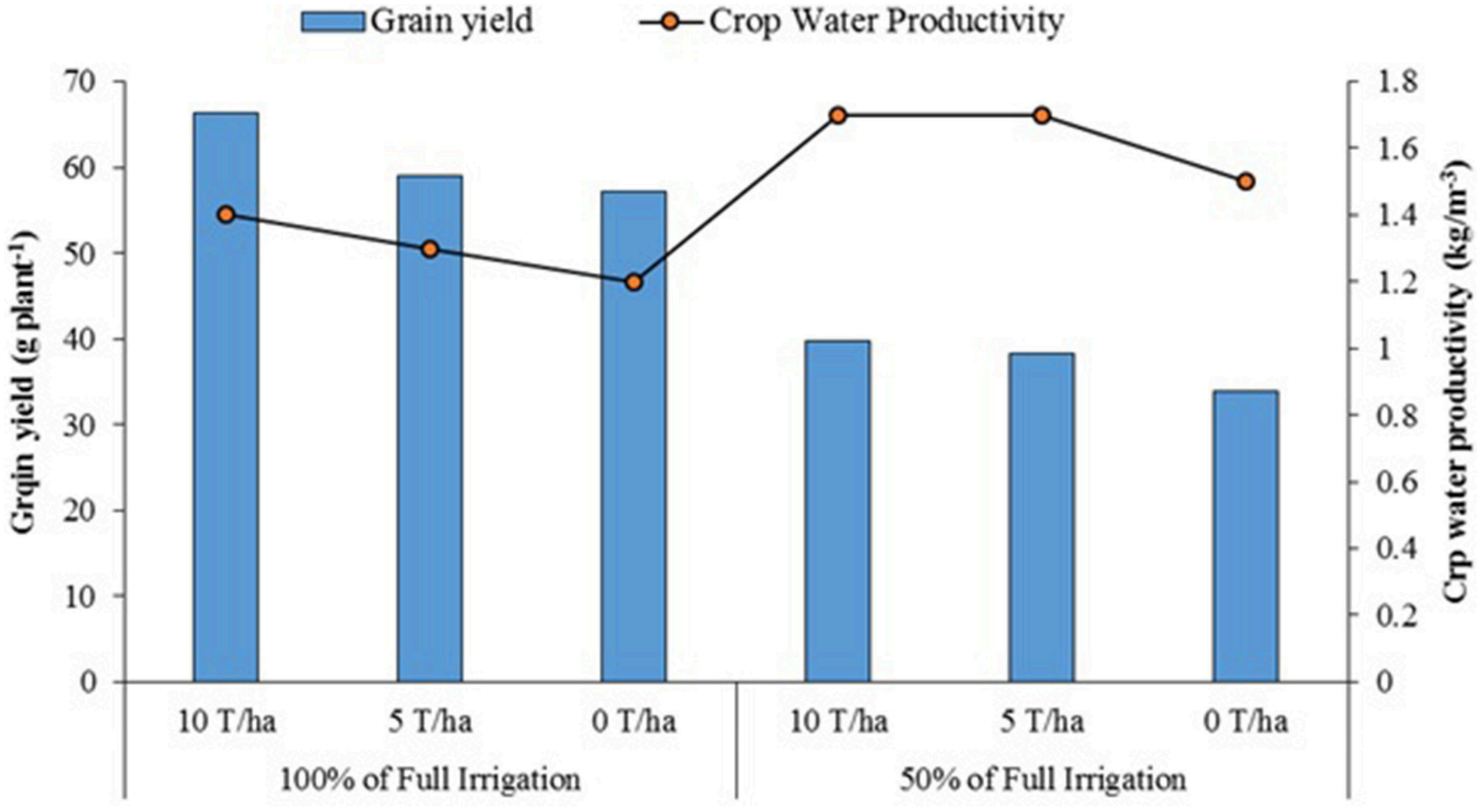
**Combined effect of organic amendment and deficit irrigation on quinoa yield and water productivity (Hirich et al., 2014a)**.

One of the most important factors which limits crop growth and production is water scarcity. As already discussed, studies show that deficit irrigation is a judicious strategy to increase water use efficiency and CWP in water-scarce environments. Organic amendments, on the other hand, improve the water holding capacity of soils and combing them with deficit irrigation can be a practical solution for sustainable yields as the negative effect of water scarcity can be compensated through the increased water holding capacity of the soils and there by improved availability of water and nutrients to the plants. In line with this, a study was conducted in at IAV-CHA to evaluate the combined effect of organic amendment (compost) and deficit irrigation using treated wastewater on productivity of the quinoa line (D0708) during October 2011 to March 2012 (Hirich et al., 2013, 2014a). Three levels of organic amendments (0, 5, and 10 t ha^−1^) combined with two levels of deficit irrigation (50 and 100% of full irrigation) were studied. While the highest seed yield (66.3 g plant^−1^) was recorded when quinoa was subjected to full irrigation and received 10 t ha^−1^ of compost, yield was low under water deficit conditions without organic amendment (Figure 5). The results indicated that organic amendment of 10 and 5 t ha^−1^ increased grain yield by 16 and 3% respectively, under full irrigation conditions, and by 18 and 13% under deficit irrigation (respectively). It can be concluded that organic amendment improve quinoa yields significantly under deficit irrigation conditions by improving the water holding capacity of the soils and the access to water and nutrients by the plants. Combining deficit irrigation and organic amendment also allowed maximizing the CWP.

### Quinoa fertilization

**Figure 6:**
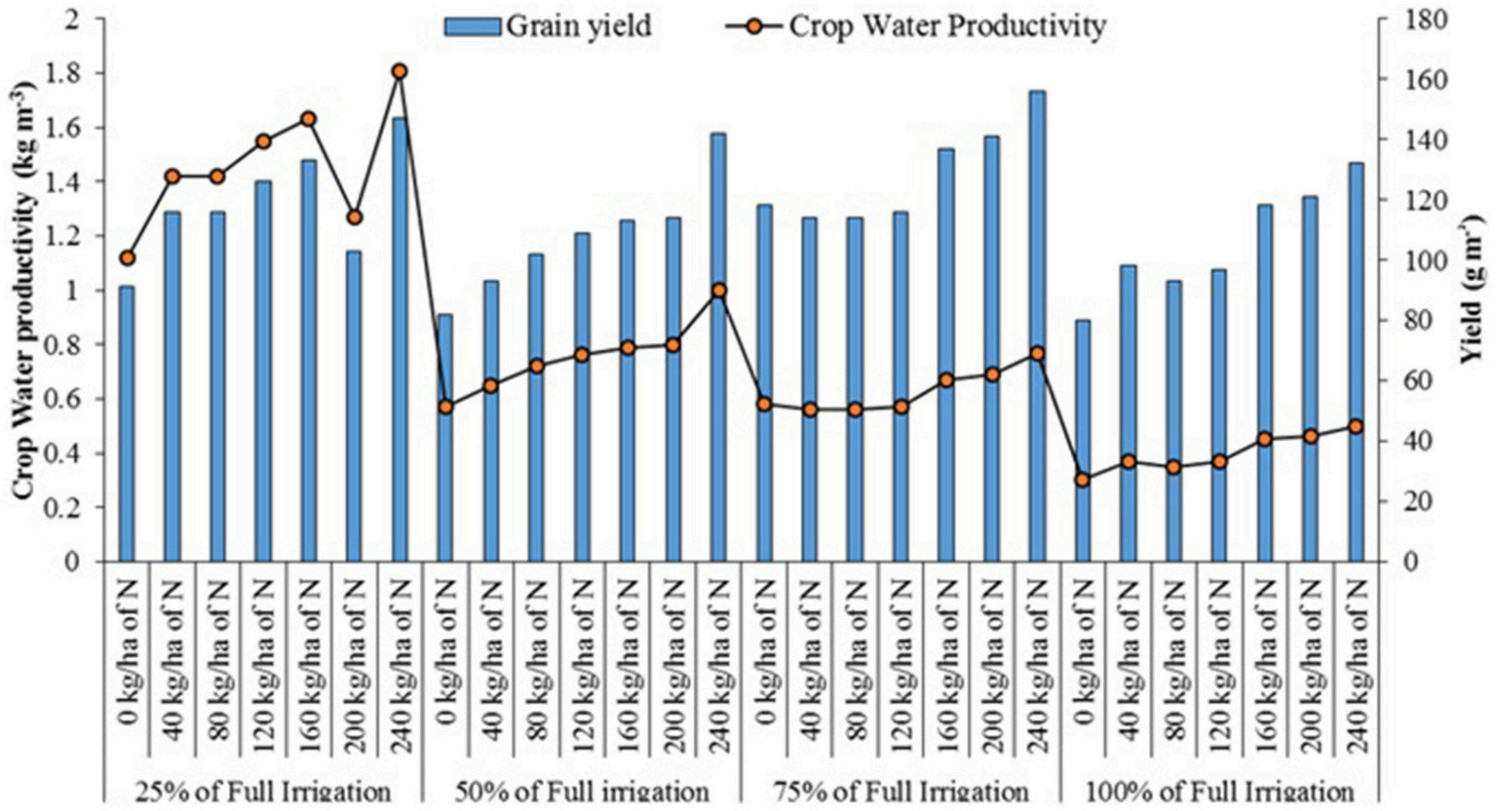
**Combined effect of water stress and N fertilization on quinoa yield and water productivity (Hirich, 2014)**.

The combined effect of water stress and nitrogen application was studied on a quinoa line (D0708) in a field trial laid out in split-plot design at IAV-CHA, during the year 2013 (Supplementary Figures 2, 4). The treatments included four levels of water stress (25, 50, 75, and 100% of full irrigation) and seven different rates of nitrogen (0, 40, 80, 120, 160, 200, and 240 kg ha^−1^) application. The results suggested that seed yield increased with increasing nitrogen supply, though the response varied with the level of water stress (Figure 6). The yield was highest in the 50% of full irrigation treatment with 240 kg ha^−1^ of nitrogen. CWP increased with higher supply of nitrogen and the degree of water stress, the value being highest in the most stressed treatment (25% of full irrigation) and 240 kg ha^−1^ of nitrogen and lowest with the full irrigation without nitrogen supply (Hirich, 2014).

In a field trial conducted in Wadi El-Natroon region, Beheira Governorate, Egypt during 2008/2009 and 2009/2010 winter seasons, Shams (2011) studied different rates of nitrogen fertilization (0, 90, 180, 270, and 360 Kg N ha^−1^) for improvement of growth and yield in sandy soils. High nitrogen fertilizer rate significantly increased yield during both the seasons. However, nitrogen use efficiency has reduced with increased rate of nitrogen application. In a pot experiment with two quinoa lines (Quinoa-52 and Quinoa-37) and two commercial varieties (Titicaca and Puno). Lavini et al. (2014) studied the effect of five rates of nitrogen application (0, 50, 100, 150, and 200 mg kg^−1^ of soil). The results showed that both the lines responded similarly to the application of nitrogen and yield has improved significantly with increased nitrogen rate.

In studies conducted by Gomaa (2013) in Egypt, quinoa plants were fertilized with ammonium nitrate (34% N) at 0, 120, 238, 357 kg ha^−1^ in combination with nitrobin (biofertilizer) or calcium super phosphate (15.5% P_2_O_5_) at 0, 120, 238, 357 kg ha^−1^ in combination of phosphorin (biofertilizer). The plants performed the best in the treatment receiving 238 kg ha^−1^ of ammonium nitrate in combination with nitrobin.

### Use of saline water

A pot experiment was carried out at the IAV-CHA to evaluate the response of quinoa (line D0708) to different levels of irrigation water salinity (1, 10, 20, and 30 dS m^−1^) (Hirich et al., 2014c,d; Lavini et al., 2014). Irrigation with water at 10 dS m^−1^ salinity has not affected seed yield significantly and yield reduction was only 9% compared to the fresh water control (1 dS m^−1^). Increasing salinity to 20 dS m^−1^ and then to 30 dS m^−1^ resulted in a significant reduction in seed yield (24 and 34%, respectively, compared to the control. Contrarily, saline water irrigation had positive effect on CWP—the most efficient treatment being the most salt-stressed treatment (30 dS m^−1^), which produced 0.8 kg for 1 m^3^ of water and the lowest being the control (0.6 kg m^−1^) (*p* < 0.001). This difference in terms of CWP can be explained in terms of the difference in water uptake which was higher in the fresh water treatment and lower in the saline treatments.

Three lines of quinoa (QM1113, QS0938, D0708) cultivated in open field were irrigated with treated wastewater with different levels of salinity in order to assess the impact on yield (El Youssfi et al., 2012). Significant differences were found in the performance of the lines—QM1113 being the most productive with an average grain yield of 6.92 t ha^−1^ at 6 dS m^−1^ salinity followed by D0708 with an average yield of 5.65 t ha^−1^ at 3 dS m^−1^. The findings indicated that quinoa yields increase with increasing salinity of up to 6 dS m^−1^ and being a facultative halophyte, salinity promotes growth but up to a certain threshold, beyond which growth and productivity start to be negatively affected. Results from these studies differ with those of Rao (2016) from on-farm trials where seed yields were maximum despite irrigation with highly saline water (15–18 dS m^−1^); and those from field experiments in Turkey where salinity stress of up to 40 dS m^−1^ did not interfere with seed and biomass yields significantly (Yazar et al., 2015); or of Razzaghi et al. (2011) who showed an yield reduction of 50% with saline water of 40 dS m^−1^ compared with fresh water.

**Figure 7:**
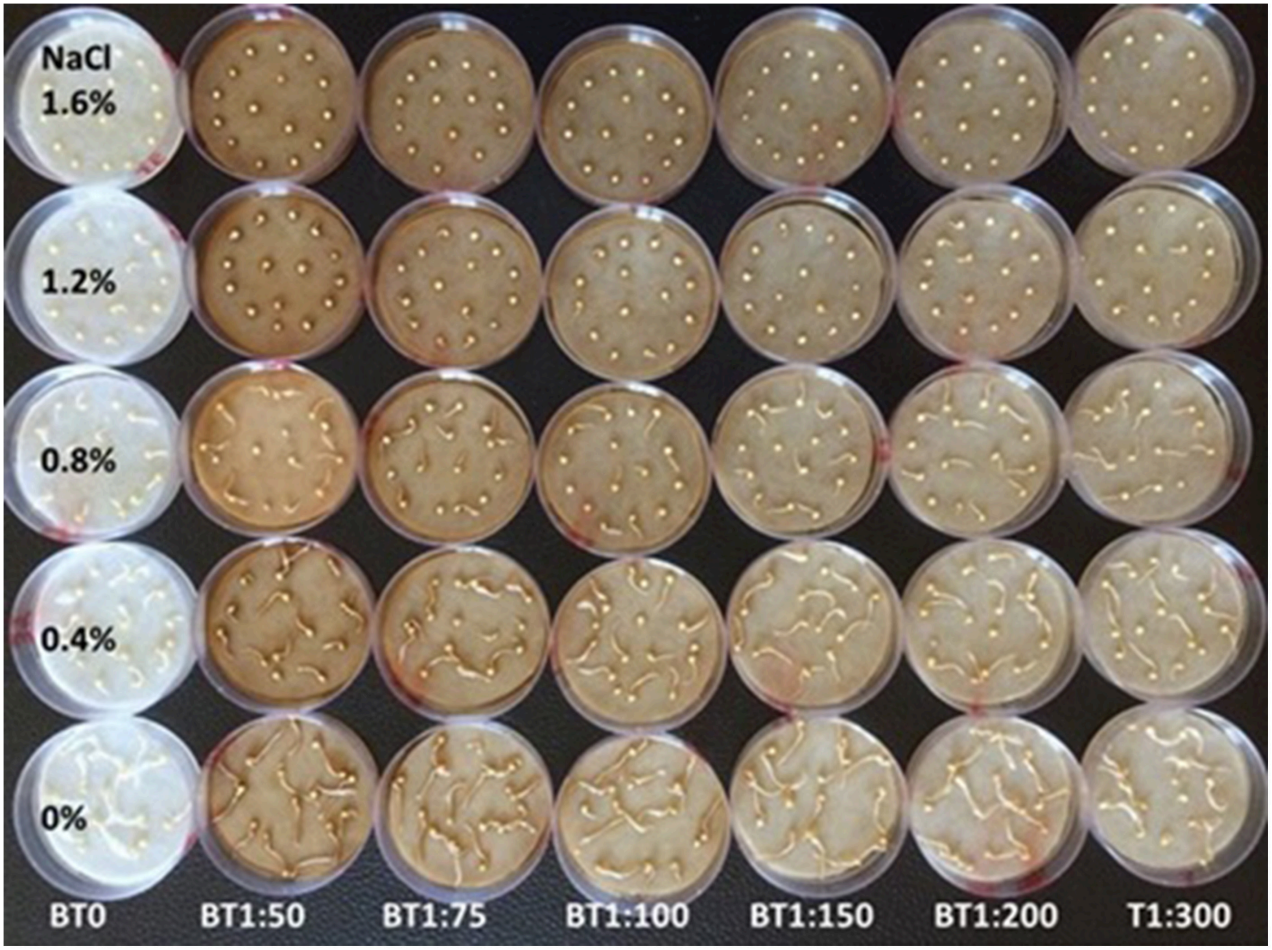
**Seed germination and seedling growth after 2 days- under different NaCl & Bontera solution Concentrations (Gill et al., 2016)**.

Simple seed germination tests were conducted at ICBA, Dubai to study the role of Bontera^TM^ (Biofertilizer and humic acid) in enhancing seed germination and in mitigating the negative impact of salinity (Figure 7). Different levels of sodium chloride of up to 50% seawater salinity (1.2% NaCl) were tested in combination with different dilutions of Bontera^TM^ (ranging from 1:20 to 1:500). The results of these tests showed that salinity in the growth medium inhibited seed germination to the extent that at 1.6% NaCl seed germination did not occur. At lower concentrations of Bontera^TM^ (dilutions of 1:500 and above) significantly enhanced seed germination and seedling growth, while higher concentrations (1:300) were inhibitory (Gill et al., 2016). Under field conditions, foliar application of Bontera^TM^ consistently showed up to 28% improvement in grain yield. It was concluded that application of Bontera^TM^ at 1–2 l ha^−1^ could reduce the chemical fertilizers application by 25%. Foliar application of Bontera^TM^ in combination with two irrigation regimes (100% ET and 50% ET) showed greater impact at 50% ET suggesting a mitigating effect for stresses like water deficiency and drought (Gill et al., 2016).

### Sowing dates

**Figure 8:**
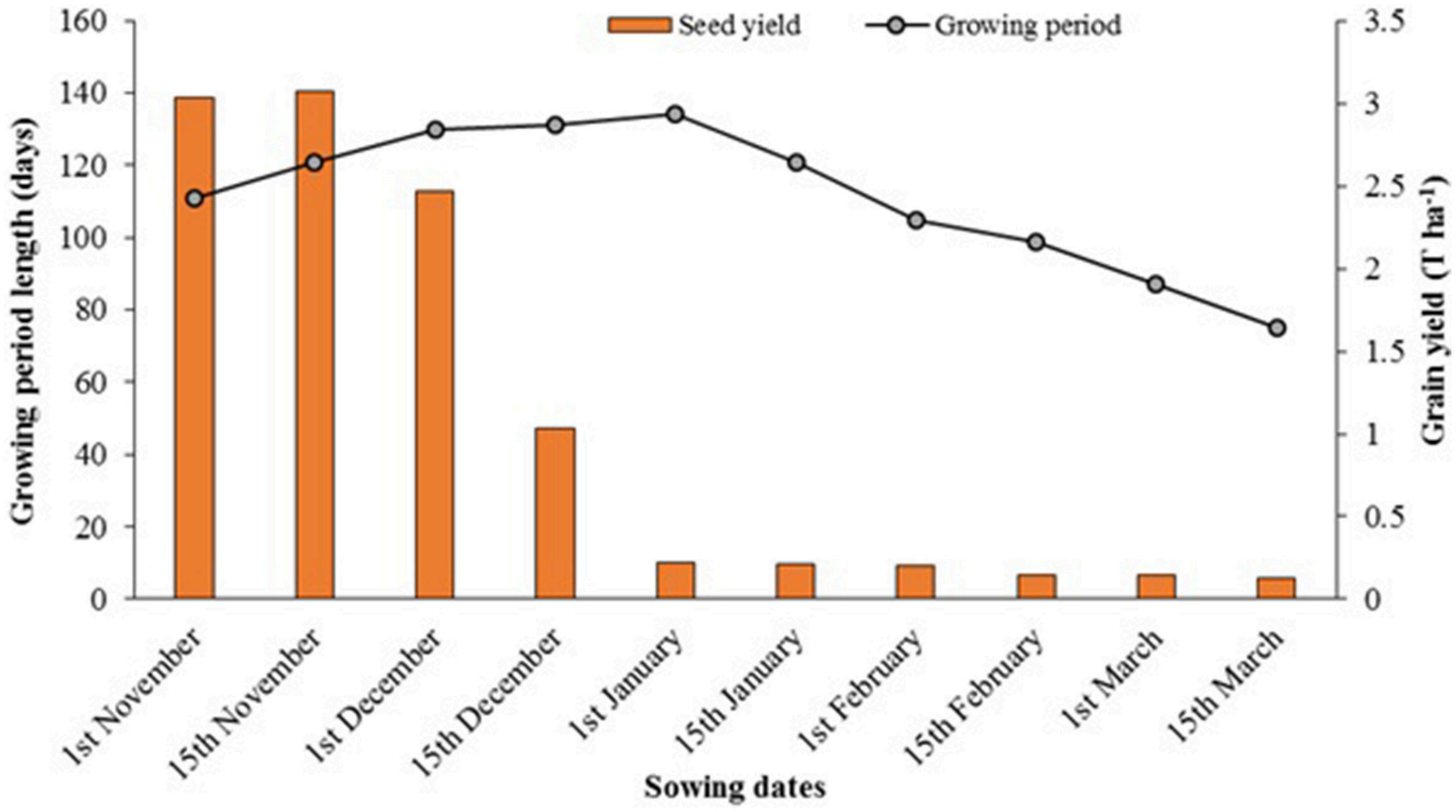
**Effect of sowing dates on quinoa yield and growing period length (Hirich et al., 2014b)**.

Hirich et al. (2014b) carried out a series of field trials at IAV-CHA (Supplementary Figure 3), to investigate the effects of 10 sowing dates—from 1st November to 15th March with 15-day intervals, on quinoa performance. The study showed that sowing dates affected growth and productivity obviously due to differences in temperature, precipitation and radiation over time (Figure 8). Seed and dry matter yields were highest when quinoa was sown in November and early December. Reduced yield during the late sowing dates was explained by delay in germination due to prevailing low temperatures and occurrence of downy mildew during February and March triggered by high air humidity. The length of growing period increased from November to January and decreased from January to March. The longest growing period was when sown on 1st of January and the shortest was when sown on 15th of March.

## Constraints for scaling up

Poor germination and crop establishment are particular problems likely to be encountered with quinoa especially in saline or other marginal environments. In the trials conducted in the UAE and the FAO project countries, significantly high variability was observed in the yields of the lines/varieties. Therefore, understanding of the influence of edaphic and climatic factors on productivity in different locations is important. There is also considerable knowledge gap regarding quinoa pests and diseases, particularly in areas outside its traditional growing regions. Some of the wild relatives such as *Chonopodium murale* and C. album are common weeds in several of the countries where quinoa is introduced and they possibly harbor a number of biotic stressors that can easily migrate onto cultivated species. Introduction and scaling-up of novel crops such as quinoa to non-traditional environments also requires the study and establishment of the entire chain and building capacity of the researchers and the farmers in the basics of production and harvesting, storage and processing technologies (Rao, 2016).

## Conclusions and recommendations

In recent years, quinoa is receiving significant attention as a nutritively rich multi-purpose agro-industrial crop that can thrive in extreme soil and climatic conditions. Faced with the challenge of increasing the production of high-quality food to feed the growing population, quinoa offers an excellent alternative to ensure food and nutrition security in marginal environments in the MENA as well as other regions across the globe. In the MENA region, while many countries have recently initiated work on quinoa, Morocco and the UAE have made significant advances toward introducing the crop in the local production systems. In Morocco, IAV HASSAN II Institute has been studying quinoa since 2000 as a drought and salinity-tolerant alternative crop to contribute to the food and nutrition security of people in the mountainous areas. As part of this program, quinoa has already been introduced to a dozen sites across the country and each year, the number of quinoa growers' is increasing all over the regions, especially Oujda, Benslimane, Fes, Boulmane, and Marrakech. However, quinoa yields over the years remained unpredictable and very low, averaging between 1.2 and 1.4 t ha^−1^, while the maximum attainable yield can be up to 8–10 t ha^−1^. A range of factors such as the choice of cultivars, optimal sowing date and nutrient management were suggested to affect the production. Constraints which still need to be overcome include crop stand establishment, sensitivity to high temperatures and salinity, weed control and saponin removal. Furthermore, there is also a need to design a product marketing strategy and raise awareness among farmers and the government agencies about quinoa's potential as a stress-tolerant alternative crop for marginal environments.

In the UAE, ICBA has been investigating quinoa since 2006 as an alternative crop for salt-affected areas. Significant progress has been made in identifying salt-tolerant lines combined with high yield potential. These lines are now being further evaluated for their yield potential in several countries including Uzbekistan, Tajikistan and Kyrgyzstan in Central Asia (through the project “Cross-regional partnerships for improving food and nutritional security in marginal environments of Central Asia” funded by the Islamic Development Bank); and Yemen, Jordan, and Egypt in the MENA region [as part of the collaborative project “Adaptation to climate change in West Asia and North Africa marginal environments through sustainable crop and livestock diversification” funded by the International Fund for Agricultural Development (IFAD), OPEC Fund for International Development (OFID)]. As with any other new crop, one of the key factors for successful introduction and establishment of quinoa under the novel climates will be the identification of appropriate planting material. It is therefore important to study the adaptation and yield potential of several quinoa genotypes from different provenances to select the most promising genotypes suitable for the local agro-climatic conditions. Information on these aspects as well as the economic assessment of the profitability of quinoa cultivation is essential, especially when planted under sub-optimal growth conditions with low-quality water in the targeted countries. ICBA's future research on quinoa focuses on developing best practices in relation to the production, management under diverse farming systems and agro-ecological regions, and on utilization and marketing of the produce, besides fostering knowledge transfer, strengthening competencies and exchange of experiences among quinoa researchers within the MENA and Central Asia regions.

It is well known that climate change is rapidly degrading the conditions of crop production. Salinization and aridity are forecasted to increase in most parts of the world, especially in the MENA region. As a consequence, new stress-tolerant genotypes of the existing crops or new and alternative crops or species must be identified and used for future food security. Stress-tolerant crops such as quinoa offer major opportunities due to their comparative advantage over the staple food crops in terms of tolerance to harsh growing conditions. The results presented in this paper have indeed shown that quinoa maintains productivity in rather poor soils and under water stress conditions and high salinity. Moreover, quinoa seeds are an exceptionally nutritious food source, owing to their high protein content with all essential amino acids, lack of gluten, high mineral content (e.g., Ca, Mg, Fe), and health-promoting compounds such as flavonoids. Higher yield do not guarantee quinoa's success in the region and to be successful it must fit in the current cropping patterns, farming systems and prove its worth in rigorous and properly designed on-farm trials in marginal lands affected by salinity and alkalinity and in the areas where the majority of the food crops could not be produced economically. For scaling up and large scale adoption by the farmers in the region, creating opportunities for marketing the produce are also vital. Quinoa being a new crop to the region, more efforts will be needed to create awareness for its incorporation in the social, cultural and dietary habits to increase the market demand besides strengthening the efforts to improve yields combined with nutritional quality, production, harvesting and post-harvesting practices along the value chain.

## Author contributions

RC was the corresponding author collecting data and results as well as editing results related to North Africa region. NR was providing research results conducted by ICBA. He was reviewing the paper and editing the English writing. AH contributed to this paper by designing the figures, commenting the data and compiling all gathered results. He was also revising the formatting according to Journal Author guideline. KT provided results of research undertaken in Central Asia. Butt has been involved with Dr. NR in several experiment on quinoa. SG provided results related to seed treatment in order to improve crop tolerance to salinity. AA provided results related to seed treatment in order to improve crop tolerance to salinity.

### Conflict of interest statement

The authors declare that the research was conducted in the absence of any commercial or financial relationships that could be construed as a potential conflict of interest.

## Supplementary material

Supplementary Figure 1 **Quinoa panicle variability in terms of color and panicle volume**.

Supplementary Figure 2 **Trial on combined impacts of water stress and nitrogen application carried out in Agadir, Morocco**.

Supplementary Figure 3 **Sowing date trial carried out in Agadir, Morocco**.

Supplementary Figure 4 **Quinoa harvest within field trial on combined impacts of water stress and nitrogen application carried out in Agadir, Morocco**.

Supplementary Figure 5 **Field trial about responses of seven quinoa accessions to several irrigation levels**.

Supplementary Figure 6 **Genetic and morphological variability of several quinoa accessions**.
