# Supplementary figures and images for: Quinoa for Marginal Environments: Toward Future Food and Nutritional Security in MENA and Central Asia Regions
Source: Front Plant Sci. 2016 Mar 29;7:346. doi: 10.3389/fpls.2016.00346 (PMC4810016; doi:10.3389/fpls.2016.00346)

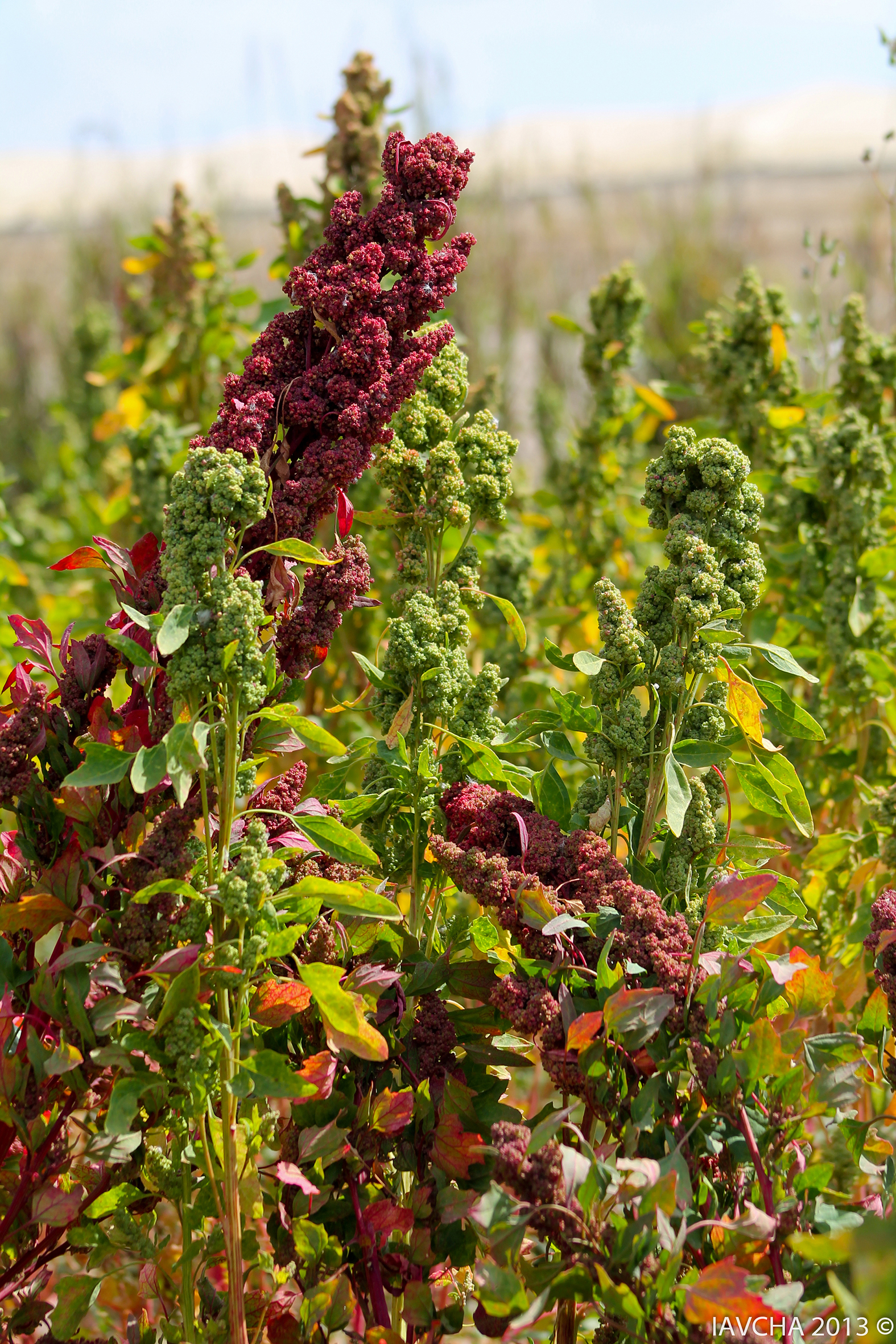

Supplement: Supplementary Figure 1 — Quinoa panicle variability in terms of color and panicle volume. [file Image1.JPEG]

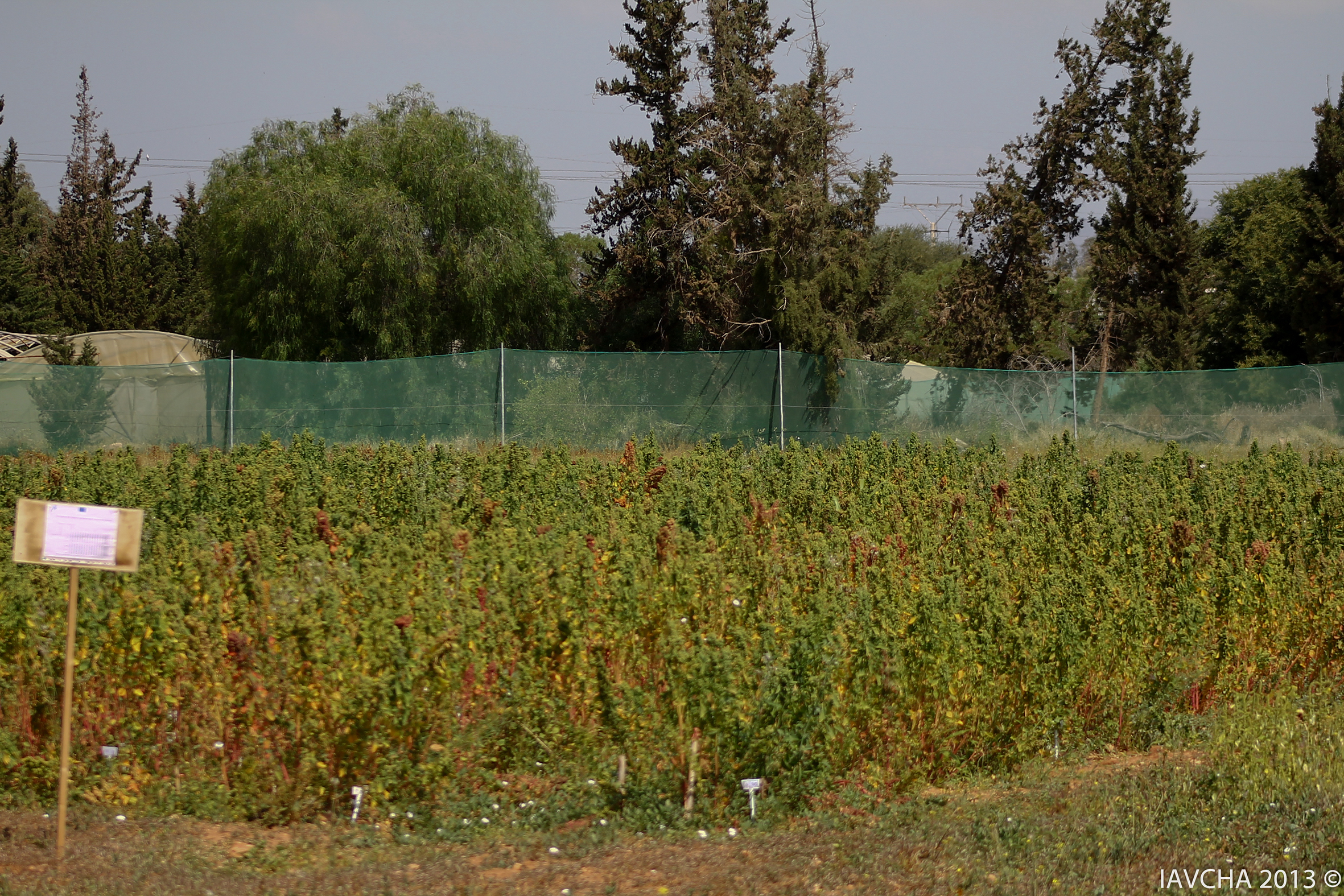

Supplement: Supplementary Figure 2 — Trial on combined impacts of water stress and nitrogen application carried out in Agadir, Morocco. [file Image2.JPEG]

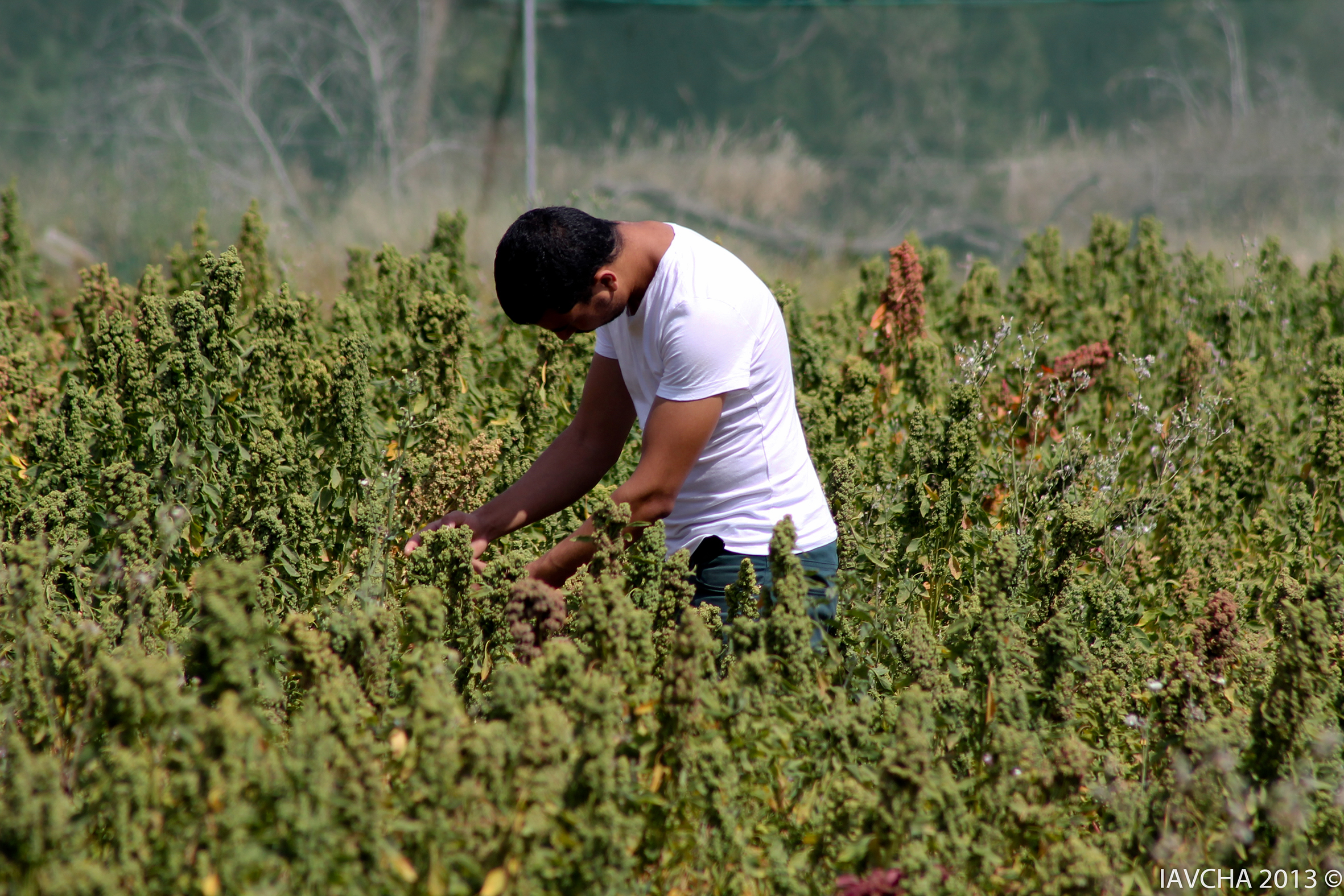

Supplement: Supplementary Figure 3 — Sowing date trial carried out in Agadir, Morocco. [file Image3.JPEG]

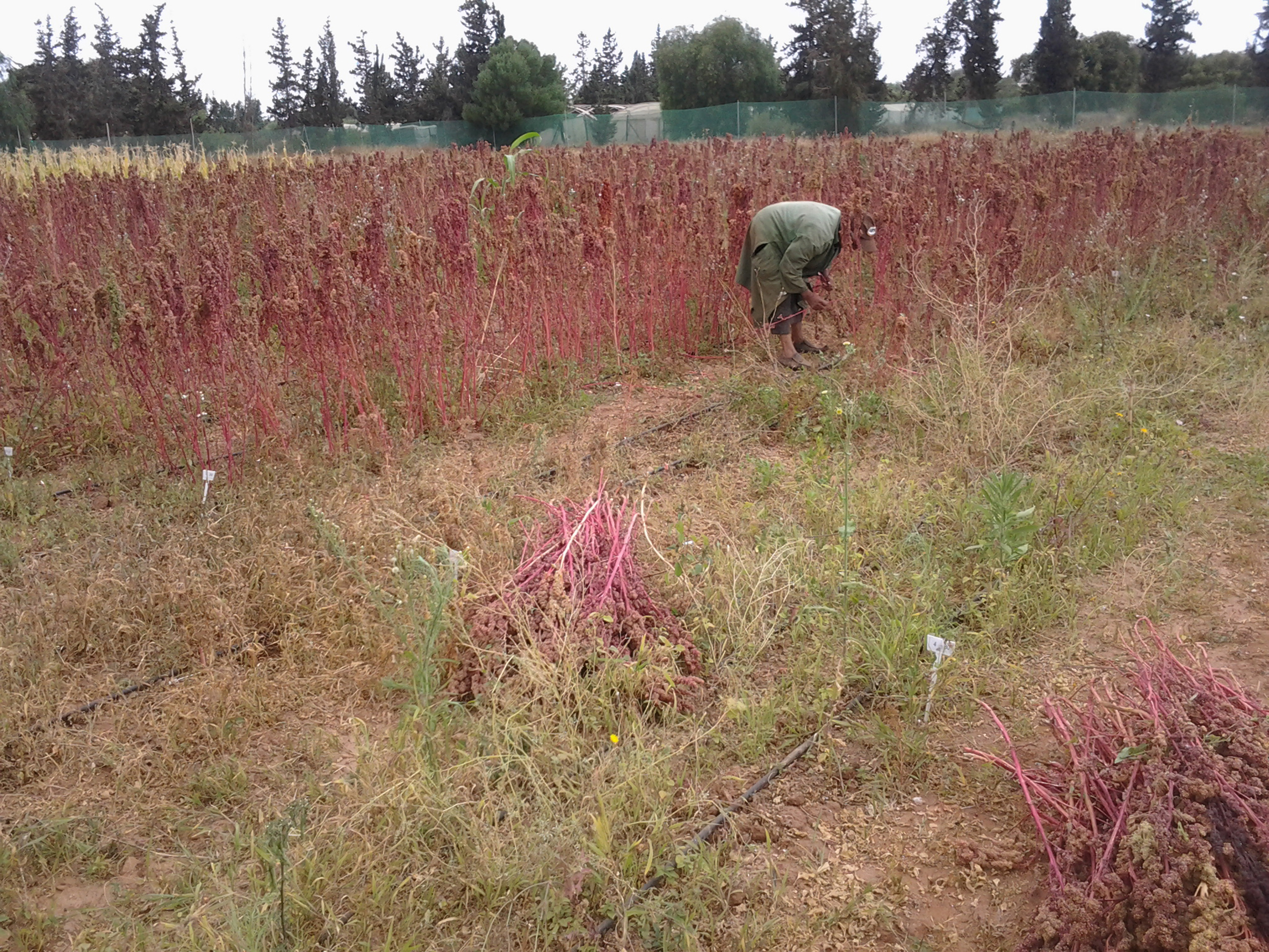

Supplement: Supplementary Figure 4 — Quinoa harvest within field trial on combined impacts of water stress and nitrogen application carried out in Agadir, Morocco. [file Image4.JPEG]

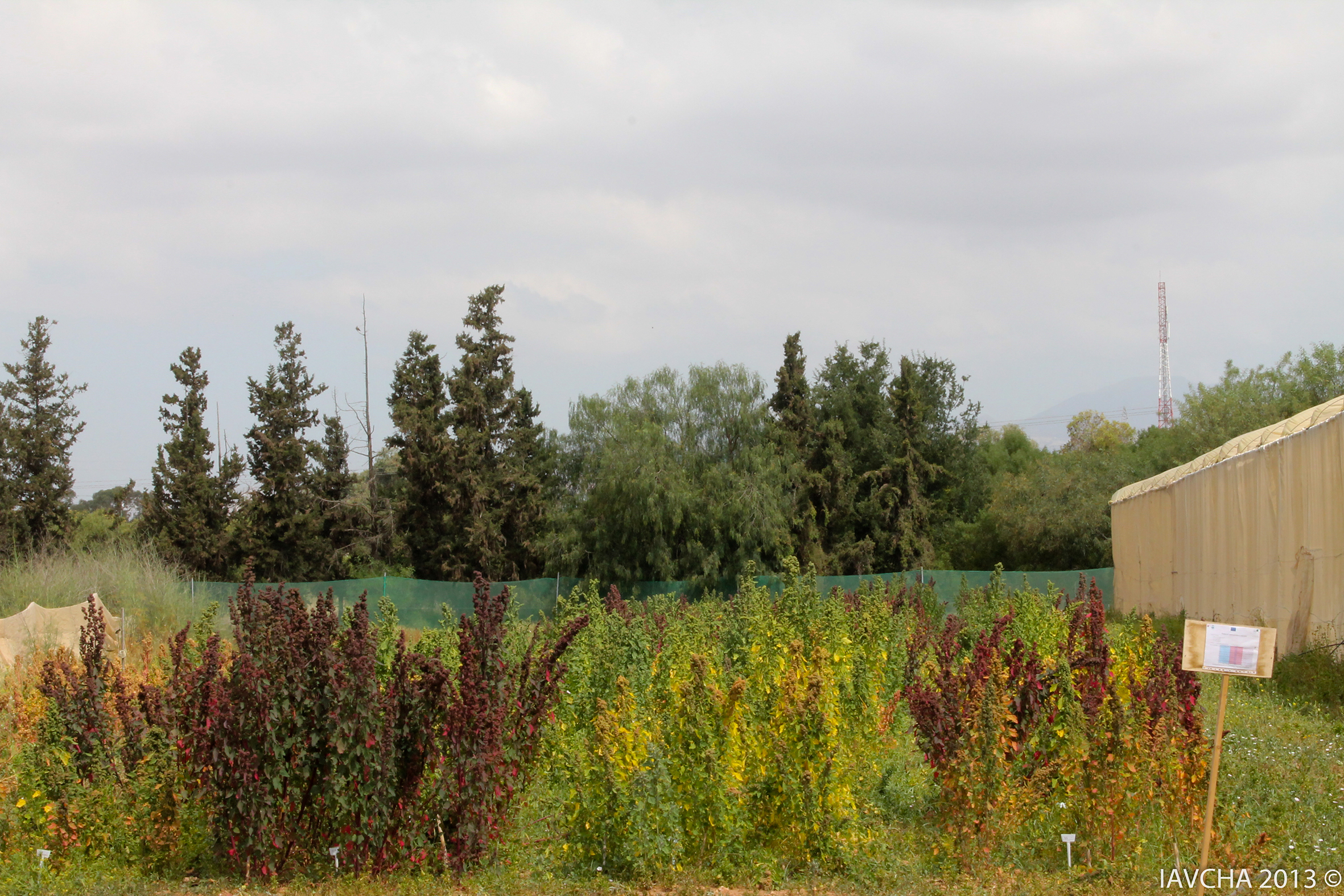

Supplement: Supplementary Figure 5 — Field trial about responses of seven quinoa accessions to several irrigation levels. [file Image5.JPEG]

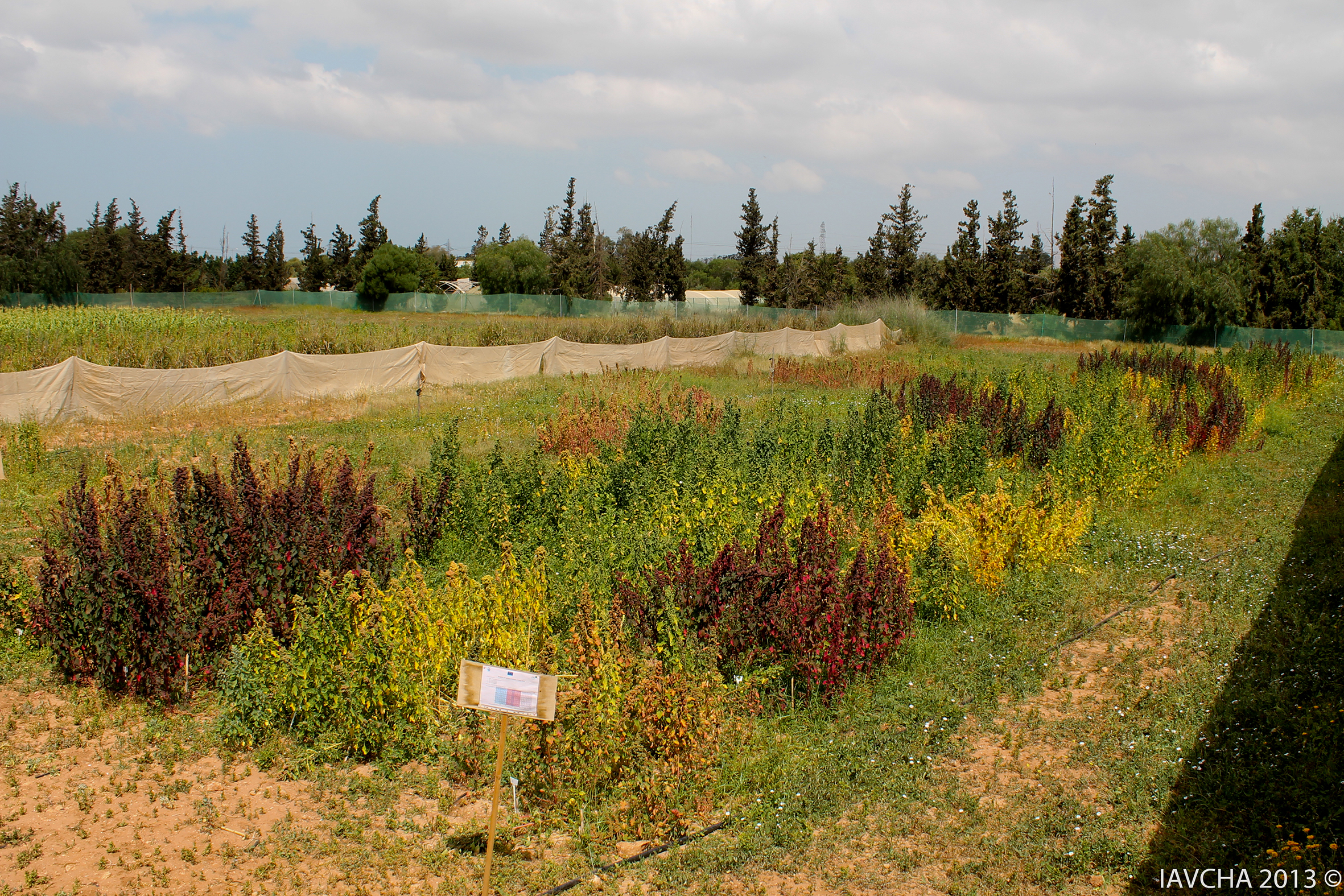

Supplement: Supplementary Figure 6 — Genetic and morphological variability of several quinoa accessions. [file Image6.JPEG]
